# Teaching Games for Understanding Intervention to Promote Physical Activity among Secondary School Students

**DOI:** 10.1155/2018/3737595

**Published:** 2018-08-09

**Authors:** Min Wang, Lijuan Wang

**Affiliations:** ^1^Shanghai University of Finance and Economic, Shanghai, China; ^2^Shanghai University of Sport, Shanghai, China

## Abstract

**Objectives:**

This study investigated the effectiveness of Teaching Games for Understanding (TGfU) intervention on the moderate-to-vigorous physical activity (MVPA) levels of students from Grades 9 and 10.

**Methods:**

A total of 118 students from four classes (two TGfU groups and two technique groups) participated in this study. Accelerometers were used to measure the MVPA time of students, and formal interviews were conducted to identify the factors that contributed to the MVPA level of students in TGfU classes.

**Results:**

The results reveal that the TGfU [*t* (1, 57) = - 11.622,* p* <.001, and* d* =.841] and technical [*t* (1, 61) = -4.232,* p* <.001, and* d* =.236] group exhibited significantly improved MVPA levels in intervention phase. During the intervention period, the MVPA time of the TGfU group (M = 20.26, SD = 3.74) was significantly longer than that of the technique group [M = 17.62, SD = 3.37,* t* (1, 118) = 4.023,* p* < .001, and* d* = .35]. Moreover, in the TGfU classes, boys (M=21.476, SD =.719) spent significantly more time engaging in MVPA than girls (M = 19.135, SD = .645) [*F* (1, 57) = 5.807, p = .019, and* η*^*2*^= .09]. However, no significant differences were determined between the MVPA levels of high- and low-skilled students. Data gathered through interviews suggested that the nature of the games, the small-sided team, and the freedom and enjoyment experienced by the students through games may explain the high MVPA levels observed in the TGfU classes.

**Conclusion:**

TGfU intervention can potentially be used to promote physical activities and attain the recommended MVPA time in PE classes (50% class time).

## 1. Introduction

Physical activity (PA) is a key component of a healthy lifestyle for the youth [1, 2]. Several surveys have revealed that Chinese children and adolescents live nonactive lifestyles because of the ongoing pressure to obtain scholastic achievements [3, 4]. Such findings have led to numerous researchers recommending school-based physical education (PE) interventions due to their effectiveness in increasing the PA levels of students [5, 6]. The United States Department of Health and Human Services (USDHHS, 2010) recommends that PE classes engage students in moderate-to-vigorous PA (MVPA) for at least 50% of the class time [7]. However, the MVPA levels of Chinese students in PE classes have never been analyzed and PE classes in the US and Europe often failed to generate sufficient MVPA levels for students [8–13]. By summarizing the studies on PA in elementary, middle school, and high school PE classes, the two reviews concluded that elementary school students spent 34.2% of class time engaging in MVPA, whereas middle and high school students spent approximately 27%-47% [9, 10]. However, due to the difficulty of increasing the frequency or duration of PE classes within existing school programs, Slingerland and Borghouts (2011) proposed the modification of teaching strategies and programs to increase the PA levels of students [13].

### 1.1. Teaching Games for Understanding (TGfU) Model and Student PA Level

Over the past few decades, PE studies have begun reflecting the limitations of traditional technique-based teaching approaches [14]. These limitations include the insufficient consideration of the contextual nature of games, an emphasis on performance, which limits the students' success, the implementation of inflexible techniques, the poor problem-solving capacities exhibited by the students, and the students' insufficient understanding of the games [14, 15]. To address these issues, Bunker and Thorpe (1982) developed the TGfU model, which integrates tactics and skills into games played in class. This model proposes the use of games, as they facilitate overcoming limitations by situating skill learning in a specific context, thus enabling the understanding of games and the development of tactical knowledge and improving problem-solving abilities through skill execution and decision-making [14–16]. Moreover, the TGfU model focuses on student learning in game education in terms of understanding game appreciation, tactical awareness, decision-making, and skill execution [16]. In contrast to the technique approach, TGfU begins with a modified game to ensure that all children can play and gain valuable insights. Learning is also guided by asking students several questions that concentrate on how children focus on several tactical aspects of a game (e.g., where goalkeepers must position themselves to stop the ball). Game-related activities are then followed by opportunities to try potential solutions.

Although TGfU primarily aims to improve the students' game performance and stimulate their learning interest, researchers have speculated on how this model promotes student PA levels. First, TGfU classes allocate a significant amount of time to the playing of games thus directly promoting PA among students [10, 16–18]. Second, TGfU classes adopt modified and small-sided games, thereby minimizing opportunity for inactivity [19]. Third, researchers have determined that like TGfU, other pedagogical models can efficiently increase PA levels within the context of a PE class (e.g., Sport Education model and SPARK program) [20–23]. Harvey and Jarrett (2014) reviewed TGfU research and noted insufficient evidence that supports the ability of this model to promote students to reach the recommended PA level [24]. Consequently, three studies have been conducted to analyze whether the TGfU model has actually increased MVPA time in PE classes [25–27], with inconsistent results. On the one hand, Dania et al. (2017) analyzed the effect of TGfU intervention on the PA of 91 Grades 3 and 4 students and determined no significant differences in PA behavior between the baseline and the TGfU intervention period [25]. On the other hand, Smith et al. (2017) compared the effects of the TGfU model and a direct instruction model on the MVPA of 72 boys and girls aged 11-12 years and found that boys reached significantly higher levels of MVPA in the TGfU classes than in the direct teaching groups, whereas no significant differences can be observed for girls [26]. Moreover, Harvey et al. (2015) determined that soccer units have utilized TGfU to increase the MVPA levels of middle school students in PE to the recommended levels [27].

## 2. Gender, Motor Skill Competency, and Student PA Level in PE Classes

Several variables, such as gender and motor skill competency, have been reported to influence the MVPA time of students in PE classes. Some studies have reported that boys are more active than girls [10, 19], whereas other studies have indicated the opposite [28–30]. Several studies have observed no difference in activity based on gender [31–33]. Moreover, other studies have compared students' MVPA with high skill and low motor skill levels in PE classes and achieved mixed results [20, 34, 35]. Li and Jr (1993) investigated the effect of PE on the fitness load of 72 secondary school students with high, moderate, or low skill competency and revealed that the highly and moderately skilled students achieved the fitness load more frequently than their considerably low-skilled colleagues[34]. Spessato, Gabbard, and Valentini (2013) investigated the PA levels of 264 children aged 5-10 years with different motor competence during PE classes and found that children with higher motor competence are considerably more active in PE classes [35]. However, Hastie and Trost (2002) analyzed the MVPA of 19 boys aged 12 years during a 22-lesson season of floor hockey and observed no significant differences in MVPA based on game skill levels. This discrepancy may be associated with different PE activities, PA level assessments (e.g., observation, heart rate monitoring, and accelerometer), teaching strategies, and the age of the subject [20].

## 3. Research Purpose

The first purpose of the present study is to determine how much of a TGfU class time students spent engaging in MVPA by using objective measures and to determine whether the TGfU model can more efficiently increases the MVPA time than the technique group. The TGfU model focused on ball games and tactics, whereas the technique group used skill teaching. The TGfU model is hypothesized to increase the MVPA time more than the technique group. Given that several variables can affect the MVPA time of students in PE classes [10, 19], the second purpose is to analyze how gender and skill levels influence MVPA levels during TGfU. The third purpose of this study is to explore the factors that determine student MVPA levels during TGfU classes by conducting interviews with teachers and students.

## 4. Research Method

### 4.1. Participants and Settings

Using convenience sampling, a high school in Shanghai City, East China, was selected to participate in this study. The study protocol was approved by the Ethics Committee of the Shanghai University of Sport and permission to conduct the study was obtained from the principal of the participating school. High school students from Grades 9 to 11 were selected for the physical, cognitive, and emotional maturity, all of which could possibly enhance their engagement in TGfU classes [36]. The participating high school is a medium-sized school with approximately 800 students across 24 classes. It is located in an urban area and receives government grants. A cluster randomization approach was used to allocate two Grade 9 and two Grade 10 classes to the TGfU and technique group; Grade 11 students declined to participate, citing their tight schedule due to the upcoming university entrance examinations. Consequently, two classes were taught using the TGfU model and two classes were taught using the traditional technique-based approach. None of these students were taught with the TGfU model in PE classes before. Informed consent form was distributed to all 146 students prior to data collection and 17 students declined to participate in the study, which resulted in 129 samples in this study. The students who did not provide informed consent were excluded from the test but remained part of the PE classes due to school requirements. Classes met two days a week, with each lesson lasting 40 minutes.

As per the provisions of the school, this intervention study was required to teach one of the sports listed in Shanghai's high school PE guideline (i.e., basketball, volleyball, and soccer). Basketball was selected for its popularity among both boys and girls in China. The two instructors who participated in the study were male and female, respectively, were in their 30s, and had over 10 years of teaching experience in basketball. These instructors learned the TGfU model through a PE teacher training program and used their training in their teaching for four to six years. Each instructor taught one TGfU group and one technique group. Then, they switched instructional approaches between the two grades to minimize teacher bias. A prestudy workshop, which was provided by a university teacher-educator who taught courses on PE pedagogies, directed the instructors to use the two models. The workshop, which lasted for three weeks and consisted of six one-hour classes, included discussions on the TGfU approach and TGfU research. Classes 1 and 2 introduced theoretical knowledge of TGfU and the differences between TGfU and other technique-based approaches. Class 3 required the two instructors to discuss videos of TGfU instruction that they had been asked to view. After the observation, the instructors were required to summarize the characteristics of the TGfU model as seen in the teaching performance of PE teachers in the videos. In Class 4, the teacher-educator demonstrated a 40-minute TGfU class with 37 Grade 10 students, focusing on the use of give-and-go to shoot in games. Finally, the two instructors were required to teach two 40-minute basketball classes using the TGfU and technique-based approaches with two groups of Grade 9 students. After the sessions, the teacher-educator presented the instructors with comments and suggestions on their teaching. The research purposes were not disseminated to the teachers and students avoid influencing the teaching behavior of the teacher. The models were introduced and compared and the lesson plans were discussed and written by the researcher and instructors.

### 4.2. Instruction

#### 4.2.1. Technique Teaching Model

A six-week (from April to May 2017), twelve-lesson (two lessons each week) intervention was adopted based on one unit of sport, excluding student assessment. The technique teaching intervention comprised skill teaching (e.g., passing, receiving, and dribbling) and two final game classes. Each lesson had a similar format, typically beginning with 5-10minutes of warm-up, followed by 20-25 minutes of skill instruction and practice and 5 minutes of cool-down or review. During the skill instruction and practice phases, the teacher initially demonstrated and explained the basketball skills and then required students to practice in drills, either alone or with a partner. Thereafter, the teachers provided skills feedback or asked a few questions concerning skill performance. Similar drills were repeated throughout the study. Sometime a game was provided to students in PE classes.

#### 4.2.2. TGfU Model

The TGfU intervention focused on tactical problems such as maintaining ball possession, using space in the attack, attacking the basket, and winning the ball. Each lesson in the TGfU approach focused on teaching the tactical elements of the game through modified games. Turner and Martinek (1992) summarized the structure of a TGfU lesson as follows: (1) the teacher sets up the game form, (2) the teacher observes play or practice, (3) the teacher and students investigate the tactical problems and potential solutions (game-related practices), (4) the teacher observes the game, (5) the teacher intervenes to promote the necessary skills skill (whenever necessary), and (6) the teacher observes the game and intervenes to teach. Following this teaching procedure, the lesson begins with a modified basketball game forms (e.g., 2 versus 2 games) [37]. The teacher observed the game and investigated tactical problems by stopping the game and asking questions to students, thereby encouraging them to think about the objectives of the game and what their main goals were before. They returned to the game. Subsequently, the teacher stopped the game and taught game principles based on how the students performed. The relevant skills (e.g., passing, dribbling) were taught and practiced as well. Finally, each lesson ended with a concluding game. Instruction steps of two groups were shown in Table 1.

### 4.3. Intervention Verification

A validation protocol developed by Turner and Martinek (1992) was used to measure the characteristics of each instruction approach and to validate the two approaches [37]. The treatment validation instrument required the coder to judge each lesson based on the following criteria: (1) the students spent most of the lesson (i.e., over 50%) engaged in games or in game-related situations, (2) the students spent the lesson learning specific skills taught by the teacher before playing a game, (3) the teacher started the lesson with skills instruction, (4) the teacher intervened in game play or game-related practices to explain strategies to students, (5) the teaching was based on observations of an initial game or game-related situations (e.g., 3 vs. 3 games), (6) lesson emphasized skill teaching, and (7) the major emphasis of the lesson was tactical instruction in games or similar practices. The responses were given using either “yes” or “no.” Three items (i.e., items 2, 3, and 6) were used to validate the technique-based approach, whereas the other four items (i.e., items 1, 4, 5, and 7) were used to validate the TGfU approach.

A two-hour training on using the validation protocol was provided to the PE teacher education student. The coder and researcher had previously watched two recorded lessons, including one TGfU lesson and one technique-based lesson, and independently completed the validation protocol with three items for the technique teaching and four items for the TGfU teaching. Thereafter, the results were compared, with interobserver agreement reaching 100%. A total of 10 lessons (i.e., five TGfU and five technique-based lessons) were randomly sampled to ensure that the instructional approaches were used appropriately. The coding results showed that all percentages of “yes” obtained for items 2, 3, and 6 through the five technique classes were 100%. The percentage of “yes” obtained for items 1, 4, 5, and 7 through the five TGfU classes was also 100%. It supported the validity of using technique-based and TGfU approaches.

### 4.4. Measures

#### 4.4.1. MVPA

Student MVPA was assessed using the Actigraph GT3X Activity Monitor, a valid and reliable tool for assessing the MVPA of children and adolescents in field settings [38]. A one-second epoch was used to avoid underestimating short periods of high-intensity activity; specific cutoff points for Chinese children and youths aged 9-17 years were used to determine activity level thresholds, thereby defining MVPA as counts per minute ≥ 2800 [39]. To measure the MVPA in each class, the participants were required to wear accelerometers throughout all 12 lessons. They were given instructions on how to wear and use the accelerometers before the treatment period began. The accelerometers were distributed to the students as the teacher took attendance. The accelerometers were fastened to the right hipbone by an elastic belt and were worn beneath their clothes for the duration of the entire lesson and were returned at the end of class. A research assistant helped check whether the accelerometers were worn properly and monitored the students to ensure that they did not take the accelerometers off during the PE classes. After class, the movement counts were uploaded to a personal computer, and raw accelerometer counts were converted into minutes spent for MVPA per class. The valid wearing time of an accelerometer was defined as being 100% of PE class time. The time taken for MVPA, Light PA (LPA), and sedentary behavior during lesson time was divided by the total lesson time to determine MVPA, LPA, and sedentary percentage time per class. Thereafter, the sum was divided by the total number of lessons to determine the MVPA, LPA, and sedentary time percentage of intervention period. The accelerometers were initialized with a personal computer to collect data before the PE classes began and were programmed to stop collecting the data at the end of the class.

#### 4.4.2. Skill Tests

In this study, “motor skill competence” refers to basketball-related skill competency and encompasses the fundamental basketball skills, such as passing and dribbling. The AAHPERD-BST passing and dribbling test (AAHPERD, 1984) was selected to measure the basketball skill level of the TGfU group [40]. The test was normed on 10,000 students from four age groups (i.e., elementary, junior high, high school, and college). The reliability and content, construct, and concurrent validity results provided adequate substantiation of the test as being a reliable and valid instrument.

#### 4.4.3. Interviews

Interview questions were designed for this study based on a review of the existing literature, the purposes of the study, and the opinions of experts. The interview questions focused on the perception of students and teachers on student PA levels in the TGfU classes and how such level was achieved. The teacher and student interview questions included two open-ended questions. (1) "What do you think about your/your students' PA levels during the TGfU classes? Did the level increase, decrease, or remain the same as those exhibited in previous PE classes? (2) What factors can increase or decrease your/your students' PA level during TGfU classes? How do these factors influence your/your students' PA levels? An expert in teacher education and a researcher specializing in TGfU provided comments and suggestions to improve the interview questions.

### 4.5. Data Collection Procedure

The protocol was 10 weeks long. The first four weeks (classes 1- 8) were established for the collection of baseline data, which followed the PE teaching guideline of high schools in Shanghai. The first class was an introduction of the PE lessons of this term and some fundamental movement skills. The contents of the other seven classes focused on running (one class), mat exercises (two classes), and rope skipping (four classes). The following six weeks (Classes 9-20) were designated as the intervention period, in which a basketball teaching unit was implemented. In these six weeks, the two groups received TGfU and technical teaching. The instructional focus and the modified games used in the TGfU and technique groups during the intervention period are shown in Table 2.

Prior to the intervention, the TGfU group participants were divided into low, middle, and high-skilled groups through basketball game skill tests. The teacher scored the performance of each student and the three scores were added together. Data from each test were converted into z-scores by gender and were then added to obtain the total score of each student. The test scores were not based on any normative criterion and were not meant to whether that students were indeed highly or lowly skilled.

After the intervention, 30 students with different MVPA levels (i.e., 10 with the highest MVPA level, 10 with the middle MVPA level, and 10 with the lowest MVPA level) were targeted for the following formal interview. Considering gender and grades, 20 students (12 boys and 8 girls; 10 Grader 9 students and 10 Grade 10 students; and 6 with high MVPA levels, 7 with middle MVPA levels, and 7 with low MVPA levels) were selected. The primary author and a trained research assistant interviewed the teacher and the 20 students. Each 15- to 25-minute interview session was audio-recorded with the participants' consent. The interviewees were encouraged to speak freely when responding to interview questions. If they did not provide adequate information, an explanation of the question, further specific information about the question, or clear examples were provided to guide their thinking. The office of the teacher was selected as the venue, with all interviews being conducted in Chinese. The recordings were then transcribed and translated into English by two research assistants.

### 4.6. Data Analysis

The quantitative data were analyzed using SPSS 20.0 statistical package. A listwise deletion of cases with missing data and spot outliers was also performed. Descriptive statistics, including mean time and percentage, were used to describe the MVPA level of students during the TGfU intervention. A 2 × 2 (group by time) analysis of variance (ANOVA) for repeated measure was applied to analyze the between-group (TGfU and technique teaching group) and within group (baseline and intervention) effects on student MVPA time. When the effects were significant, Tukey's post hoc tests were performed to locate the group differences and a paired-sample t-test was adopted to determine the differences between the pre- and posttests of each group. A two-way (gender × skill level) ANOVA was conducted to gauge the differences in the mean time of the MVPA of the TGfU group by gender, skill level, or gender × skill level in the data. The effect size was calculated to avoid bias of the *p* value.

The interview data obtained in the present study were analyzed using deductive content analysis using the approach proposed by Patton (2002), in which the management and analysis of the interview data involve several steps [41]. First, the interview data were prepared for analysis through the transcription and translation of the audio-recorded interviews, as performed by two research assistants. Second, the themes of the raw data were identified for each participant. The raw data themes consisted of the summary of the passage and several key words, phrases, or sentences in the interview data that conveyed a specific concept or idea. Third, the raw data themes of each participant were compared to identify the common themes (e.g., nature of the game, small-sided team, and enjoyment of the class). The first-order themes were subsequently categorized as several general dimensions (i.e., determining factors). Finally, summaries of the raw data, first-order themes, general dimensions, and categories for the participants were combined to form a hierarchical thematic structure. The names of the participants were changed (e.g., Teacher 1 and Student 1) to ensure their anonymity. The validity of the interview data was then established using three strategies: peer debriefing, member checking, and analyst triangulation [41].

## 5. Results

### 5.1. Demographic Characteristics

A total of 11 subjects were found to have missing data (seven students missed one or more PE classes and two students did not attend skill tests) or serve as outliers (MVPA time of two students in a certain PE class was less than one minute because of accelerometer malfunction) and were consequently removed, as they could potentially bias the results. Hence, the final sample used for calculations consisted of 118 students with a mean age of 16.47 years (SD = 0.68). Among the participants, 57 students (26 boys and 31 girls, mean age = 16.59 years, and SD = 0.65) were selected for the TGfU group and 61students (33 boys and 28 girls, mean age = 16.36 years, and SD = 0.68) were taught with the technique approach. Among the TGfU group, 29 students with negative total* z*-scores were labeled for analysis as “higher skilled” (M = -1.238, SD=0.640), and the remaining 28 students with positive total* z*-scores were labeled as “lower skilled” (M = 1.283, SD =0.787).

### 5.2. Effect of the Two Teaching Models on the MVPA of Students

The measures of TGfU intervention revealed that students spent an average of 20.26 mins (SD = 3.74), 10.39 mins (SD = 3.65), and 9.30 mins (SD = 1.70) in MVPA, LPA, and sedentary behavior per PE class, equivalent to 50.7%, 25.9%, and 23.4% of the lesson time.  Table 3 shows the means and standard deviations in the MVPA time of the students in the TGfU and technical and group before and after the intervention. Repeated-measure ANOVA showed significant group × time interaction for time spent on MVPA [F (1, 118) = 99.706,* p* < .001, and* η*^*2*^ = .460]. Paired-sample t-tests showed that the TGfU group [t (1, 57) = - 11.622,* p* <.001, and* d* =.841] and technical group [t (1, 61) = -4.232,* p* <.01, and* d* = .236] improved significantly from the baseline to the intervention phase. Independent sample t-tests also showed that the time spent by the two groups on MVA at the baseline exhibited no significant differences [t (1, 118) = -1.687,* p* = .068, and* d* = .31]. However, the MVPA times of the two groups at the intervention phases were significantly different [t (1, 118) = 4.023,* p* < .001, and* d* = .35]. The TGfU group (M = 20.26, SD = 3.74) had a significantly higher MVPA level than the technique group (M = 17.62, SD = 3.37).

### 5.3. Gender, Skill Levels, and Student MVPA Level in TGfU Classes

Table 4 shows the means, standard deviations, and* F*-values of the time spent on MVPA based on gender, skill level, and gender × skill level. In the basketball unit, a significant difference in gender was found in the performance of either gender [*F* (1, 57) = 5.807, p = .019, and* η*^*2*^= 0.09], as the MVP time of boys (M=21.476, SD = .719) was significantly higher than that of girls (M = 19.135, SD = .654). The study was unable to determine any significant differences between the MVPA levels of the high- and low-skilled students [*F* (1, 57) = 0.018, p =.893, and* η*^*2*^= .000], although high-skilled students (M = 20.297, SD = 3.824) exhibited greater MVPA times than low-skilled students (M = 20.259, SD = 3.747). Moreover, no significant gender** ×** skill level interactions for MVPA were observed [*F* (1, 57) = 0.934, p = .338, and* η*^*2*^= .017].

### 5.4. Factors That Determine Student MVPA Level during TGfU Classes

Two PE teachers and 20 students participated in interviews which focused on the factors that determine the MVPA of students in the TGfU classes. Most students and all the PE teachers felt that MVPA was increased in TGfU classes, consistent with the quantitative data. Several themes (i.e., the nature of the games, the modification of the games, enjoyment of the games, and the freedom provided by TGfU classes) emerged, which may explain the PA levels of students in TGfU classes.

#### 5.4.1. Nature of the Games

The nature of the games served as the first subtheme explaining the increased PA level of students. Teacher 1 (male, 38) reported that playing games offered more opportunities for students to participate in the task and explained that “…The PA level is higher in games than in skill practice… Students have to keep on moving and running to receive or throw a ball in games.” Some typical responses from students were as follows:

  It (TGfU class) is more tiring. In our previous classes, we spent most of our time just standing around, passing a ball back and forth. Now, we're always running during games because we're trying to evade defenders to pass and receive the ball and just standing around will not help with that (student 3, male, 18).
  The game is characterized by competition. We did our best to do everything to win the game like moving fast and trying to grab the ball from competitors. We also exerted more effort into the game than we did in our previous classes (student 16, male, 15).
  I felt that we are more active in TGfU classes…I have to keep on moving and running to receive or throw a ball in games, whereas most of the time, I just had to stand there and practice these skills in previous classes (student 7, female, 17).

#### 5.4.2. Modification of the Games

Except for the nature of the games, teachers and students likewise discussed how modified games provide students with more opportunities for involvement. Teacher 2 (female, 34) indicated that modifying the games according to the abilities of the students is important to improve PA. He stated that

  “At the beginning of the unit, I felt that game tactics were difficult for students to understand and apply. I wasted significant amount of time clarifying and demonstrating game tactics…So, I tried to simplify the games and found that students become more involved in the class.”

Student 5 (male, 18) also mentioned the small-sided games, stating that “Small-sided games, for instance, 2 versus 2 or 3 versus 3 games, are great…My teammate always passes the ball to me, so I had more opportunities to hold the ball and participate, which could have enhanced my PA level.” Student 12 (male, 17) also discussed how modifying the games lowered motor skill level requirements, saying “TGfU provides interesting games that are modified to only require fundamental skills. I am not exactly a skillful person, but because the games are modified, I can still be involved and that makes me more physically active.” Moreover, Student 9 (female, 16) talked about modified game rules, explaining that, “…The game rules were simplified and changed in TGfU classes. We did not have to repeatedly stop the games due to rule breaking. The modifications made to the game rules gave us more freedom and opportunities to be involved in games.”

#### 5.4.3. Enjoyment in the Games

The third important subtheme revealed by the teacher and student interviews was their enjoyment of the games. Teacher 1 (male, 38) indicated that students liked the games which promoted their engagement in classes and thus enhanced their PA level. He said “Students like games, and games excite them. They always laugh and shout in games. Participating in games made them move more actively and consume more physical energy.” Student 20 (female, 16) said “I am more involved in classes because I like games. I run actively during games and try my best to take the ball and score. This activity really consumed my physical energy.” Student 8 (male, 18) also responded “In previous classes, we just repeated the same motor skills, which was boring…of course since I like games more, was thus more active in joining them.”

#### 5.4.4. More Freedom

Finally, a small group of students reflected that the increased freedom provided in TGfU classes induced the increase in their PA level. Some typical comments were as follows:

  I am freer in games and I do not care whether I performed a skill correctly or not; I simply cooperate with my teammate and try to score (student 11, male, 16).
  In previous PE classes, I had to follow the step-by-step instructions of my teacher to properly perform the skills. Now, I can play games the way I want to, and this change allowed me to play more actively (student 14, male, 16).
  I am free in games. I run around the court, which makes me more physically active (student 1, male, 17).

## 6. Discussion

The accelerometer data in the present study indicated that the average MVPA time of the TGfU group was significantly longer than those of the technique-based group, reaching the recommended MVPA time of PE class (50% class time). This finding is consistent with previous TGfU intervention studies [25, 26] and supports the premise that the game-based teaching model could enhance the MVPA level of students [19]. However, Fairclough and Stratton (2005) reported that numerous interventions improved the student MVPA through high-intensity activities and training-like protocols that ignored the education goals [9]. Thus, they suggested that a further favorable intervention should stimulate fitness and consider wider PE focus [9]. The TGfU model was proved to develop student awareness of game tactics, improve tactical knowledge, and stimulate their interests in PE (i.e., educational focus) [42–44], implying that TGfU promoted student MVPA level, thereby providing an additional valuable component of the model (i.e., fitness stimulation). This finding is consistent with the recommendation of Fairclough and Stratton (2005) [9]. Accordingly, TGfU could reasonably and effectively increase the PA levels in PE classes.

As per the data collected from the interviews with the teachers and students, the nature of the games, the small-sided teams, the student enjoyment in class, and the additional freedom may explain the improvement of MVPA levels during TGfU classes. Previous studies have emphasized the nature of games in promoting the PA levels of students [20, 45], concluding that team games promote the highest level of MVPA because they involve large amounts of full body translocation across space and enhance the physiological load on the working muscles [45]. The enhancement of MVPA levels due to small-sided teams can also be explained by the fact that a small-sided team induces additional intense playing conditions and enables students to be actively involved [20]. In small-sided teams, students have very limited opportunities to be inactive by hiding in the outfield or becoming “competent bystanders.” Meanwhile, the relationship between student enjoyment in PE class and their PA levels during class has rarely been reported in previous studies. Only Fairclough (2003) has examined the levels of activity, enjoyment, and perceived competence during lessons, concluding that enjoyment was highest during team games, thereby engaging students in MVPA with significantly more time than individual activities [46]. The proposition supports the study's findings that the enjoyment and engagement of students in TGfU classes enhanced their MVPA levels. Finally, several students confirmed that gaining additional freedom in TGfU classes increased their MVPA levels. Traditional technique-based teaching is characterized by following step-by-step instructions provided by teachers and focusing on skill performance, thereby restricting the physical exertion of students, whereas TGfU classes enable students to freely decide and solve problems in games without requirements or restrictions on skill performance [14, 36, 47]. Hence, the TGfU model is accompanied by high levels of PA.

Moreover, these factors could have promoted the MVPA of students by stimulating their higher motivation to be involved in PA during TGfU classes. Self-determination theory (SDT) indicates that students become autonomously motivated, thereby eliciting high-quality motivation when their innate needs, such as competence, autonomy, and relatedness, are satisfied [48]. Several researchers have applied SDT in PE and determined that students who display high levels of autonomous motivation are further stimulated and motivated by PE, thereby resulting in an increase in PA levels during PE classes [49, 50]. In the present study, teachers and students confirmed that PA levels were influenced by the freedom and enjoyment they experienced through the TGfU classes and the small-sided teams. The freedom in games provided autonomy for the students whereas the small-sided games enhanced students' enjoyment and sense of being connected with other students as well as their feeling of competence, given small-sided games provided additional opportunities to be involved. The satisfaction of these three psychological needs may promote the autonomous motivation of students, thereby enhancing their MVPA time in TGfU classes.

As supported by other PE studies, boys were found to have spent more time involved in MVPA than girls during TGfU units, thereby implying that TGfU intervention was considerably more effective in increasing PA levels in boys than in girls [9, 19, 46]. This has three possible explanations: the first possible being the mixed-gender nature of PE classes. Several studies have reported that boys in mixed-gender classes tend to obtain more total practice trials and more appropriate practice trials than girls, thereby possibly assisting the former achieve higher PA levels [51]. Second, several researchers have reported that males tend to enjoy team sports more than females do, thereby increasing the PA levels of the former [52, 53]. Third, the gender differences in the MVPA time may be related to the sports taught in this intervention study. Basketball may be more attractive to boys than to girls. Hence, boys were substantially motivated to participate in the games [54].

Contrary to the findings of previous studies, no significant difference was observed in the MVPA time of high- and low-skilled students. Previous research has reported that most low-skilled students are less engaged in MVPA class time than their high-skilled peers [9, 18, 34]. The different methods used to distinguish between high- and low-skilled students from each other may account for the difference in results. This is because past studies used student self-reporting of perceived sports skill levels and classification of PE teachers [18, 34], whereas the current study determined skill level through skill tests classified with z-scores by gender. The nonsignificant difference may indicate that teachers attempted to be cautious of individual differences and provide the students with equal opportunities for optimal activity engagement using TGfU. This condition supports the strength of the TGfU model, wherein games could be modified to include students with varying motor skill levels [36].

## 7. Strengths and Limitations

The strengths of this study include the mixed-method approach, intervention validity, and the use of accelerometers for assessing MVPA. The mixed-method approach was used to evaluate effectiveness of the intervention, thereby enhancing the trustworthiness and validity of the data through method triangulation. A further strength of this study was that the lessons were planned and delivered by PE teachers who had sufficient experience with TGfU and technique-based teaching, thereby ensuring the validity of intervention. Finally, the use of objective measures of MVPA ruled out the potential for subjective biases in self-reporting.

However, the present study has several limitations. First, this study is limited by its sole utilization of the direct comments of the teachers and students when attempting to explain the students' PA levels in the TGfU classes. A further study that uses the systematic observation of TGfU teacher behavior is necessary to determine the relationship among student PA level, lesson contexts, and the behavior of teachers toward TGfU classes. Second, the data were derived from a small group of students from Grades 9 to 11 because they were deemed mature enough to participate in TGfU classes. Hence, the findings of this study cannot be generalized to primary and secondary school students, as they do not meet the inclusion criterion of this study's sample. Third, the present study was designed to determine the levels of MVPA during a basketball teaching unit over a relatively short period of time limiting the findings generalizability to other sports (e.g., soccer, volleyball, and hockey) and all TGfU classes. Future studies should test the MVPA time of students during a wide range of sports activities and over additional lessons.

## 8. Conclusion

Compared with the technique-based teaching approach, the TGfU model significantly increased MVPA time. Given that TGfU intervention has the potential to promote PA during PE classes to reach the recommended MVPA time of a PE class, hence TGfU teaching should be encouraged to improve the low MVPA engagement levels during PE classes. Furthermore, male students spent a significantly more lesson time involved in MVPA than females during the TGfU classes. Therefore, further attention should be given to girls to help increase their MVPA.

## Figures and Tables

**Table 1:** Instructional procedure of TGfU and technique teaching approaches.

Instruction steps	TGfU group	Technique group
1	The teacher sets up the game form	The teacher demonstrated and explained the basketball skills
2	Students play games and the teacher observes play or practice	Students practice in drills, either alone or with a partner.
3	The teacher and students investigates the tactical problems and potential solutions	The teachers provided skills feedback or asked a few questions concerning skill performance.
4	Similar or other games were played by students. Sometimes the teacher intervenes to promote the necessary skills skill	Similar drills were repeated. Sometimes a game was provided to students

**Table 2:** Instructional focus of TGfU and technique teaching approaches.

Lesson	Instructional Focus	
	TGfU group	Technique group
9	Tactical problem: Maintaining possession of the ball Lesson focus: passing, and receiving a pass, ball fake, pivots	Ball handling (e.g., wake-up, toss, catch and squeeze, how to hold the ball, around the waist); jump stops and pivots Lesson focus: learning of the basketball skills
10	Tactical problem: Attacking the basket Lesson focus:shooting within five to eight feet of the basket	Passing (chest pass, bounce pass, overhead pass), and receiving passes Lesson focus: learning of the basketball skills
11	Tactical problem: Maintaining possession of the ball to support teammate Lesson focus: create passing lanes	Passing on the move (forward/backward), jab step, drop step Lesson focus: learning of the basketball skills
12	Tactical problem: Creating space in the attack Lesson focus: creating passing lanes	Control dribble (high/low); Control dribble on the move (forward/backward) Lesson focus: learning of the basketball skills
13	Tactical problem: Using space in the attack Lesson focus: use the dribble for repositioning to make a pass	Dribble with different parts of hand, crossover, speed dribble Lesson focus: learning of the basketball skills
14	Tactical problem: Attacking the goal Lesson focus: Identify when there is an open lane to the basket; then dribble to drive and shoot	Shooting technique Lesson focus: learning of the basketball skills
15	Tactical problem: Attacking the basket Lesson focus: using the give-and-go to score	Spot form shooting Lesson focus: learning of the basketball skills
16	Tactical problem: Creating space in attack Lesson focus: Using pick and screen to create space	Moving without the ball/getting open Lesson focus: learning of the basketball skills
17	Tactical problem: Winning the ball Lesson focus: defensive positioning on and off the ball	jump shooting, layup shooting Lesson focus: learning of the basketball skills
18	Tactical problem: Winning the ball Lesson focus: preventing offensive team from passing, receiving passes, and scoring	Rebound skill Lesson focus: learning of the basketball skills
19	Tactical problem: Defending space Lesson focus: defending against a screen	Games Lesson focus: play games with skills learned
20	Tactical problem: Defending space Lesson focus: student-to-student team defense	Games Lesson focus: play games with skills learned

**Table 3:** Means (M), standard deviation (SD), and F value of time in minutes spent in MVPA.

	TGfU group		Technical group		
	M	SD	M	SD	F
Baseline	14.98	3.15	15.99	3.34	99.706*∗∗∗*
Intervention phase	20.26	3.74	17.62	3.37	

*Note.∗P*< .05, *∗∗P*< .01, and *∗∗∗P *< .001.

**Table 4:** Means, standard deviations, and F-value of the MVPA time of TGfU group.

	MVPA time (N =57)		
	M	SD	F
Gender			5.807*∗*
Boys	21.476	.719	
Girls	19.135	.654	
Skill level			.018
High-skilled group	20.297	3.824	
Low-skilled group	20.259	3.747	
Gender **×** skill level			.934

*Note.∗p*<0.05 and *∗∗p*<0.01.

## Data Availability

The data used to support the findings of this study are available from the corresponding author upon request.
